# Supplementary material for: Isolation and Genomic Characteristics of Cat-Borne Campylobacter felis sp. nov. and Sheep-Borne Campylobacter ovis sp. nov
Source: Microorganisms. 2023 Apr 8;11(4):971. doi: 10.3390/microorganisms11040971 (PMC10145079; doi:10.3390/microorganisms11040971)
Supplement: Supplementary file 1 [file microorganisms-11-00971-s001.zip › Fig. S2.pdf]

Figure S2: Maximum-parsimony phylogenetic tree based on nearly complete 16S rRNA gene showing the relationships between our isolates and the type strains of the genus *Campylobacter*. Bootstrap values (>70%) based on 1000 replicates are shown at branch nodes, with *Arcobacter butzleri* ATCC 49616^{T} as an outgroup. Novel strains are highlighted in bold.
