# Supplementary material for: Isolation and Genomic Characteristics of Cat-Borne Campylobacter felis sp. nov. and Sheep-Borne Campylobacter ovis sp. nov
Source: Microorganisms. 2023 Apr 8;11(4):971. doi: 10.3390/microorganisms11040971 (PMC10145079; doi:10.3390/microorganisms11040971)
Supplement: Supplementary file 1 [file microorganisms-11-00971-s001.zip › Table S1.pdf]

Table S1: ANI (lower diagonal) and dDDH (upper diagonal) among the novel *Campylobacter* strains and other *Campylobacter* species
