# Supplementary figures and images for: Isolation and Genomic Characteristics of Cat-Borne Campylobacter felis sp. nov. and Sheep-Borne Campylobacter ovis sp. nov
Source: Microorganisms. 2023 Apr 8;11(4):971. doi: 10.3390/microorganisms11040971 (PMC10145079; doi:10.3390/microorganisms11040971)

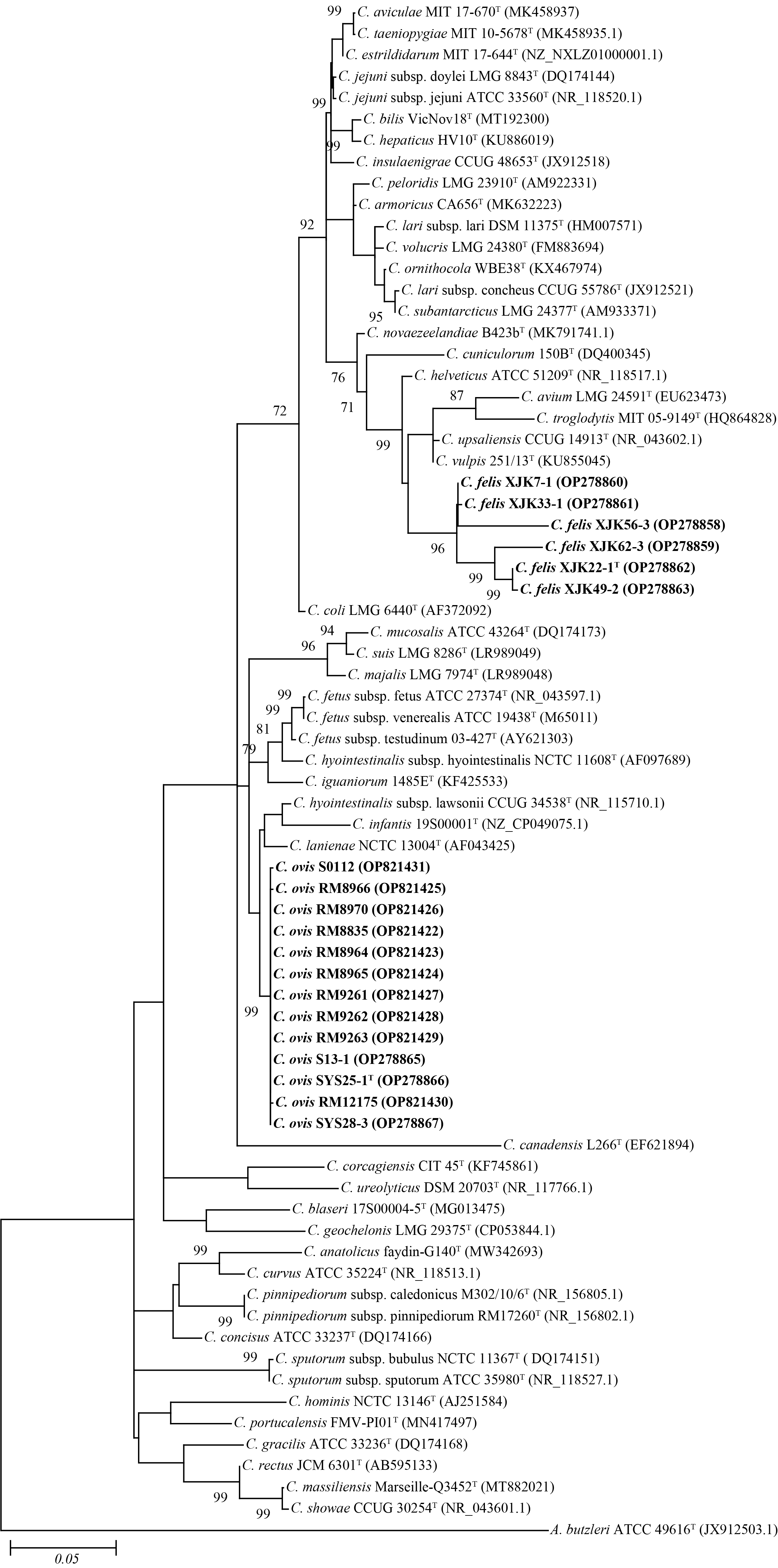

Supplement: Supplementary file 1 [file microorganisms-11-00971-s001.zip › Fig. S1.tif]

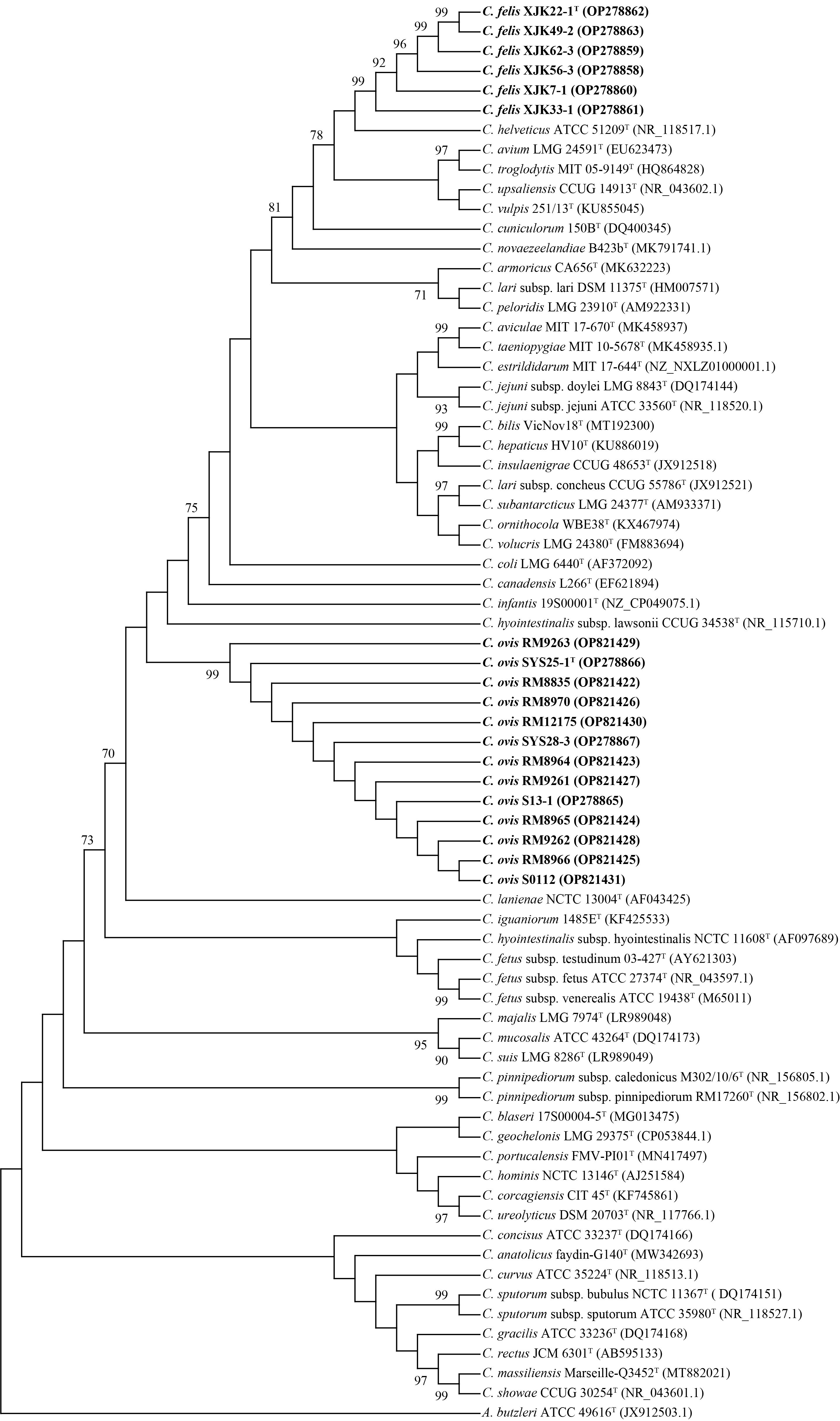

Supplement: Supplementary file 1 [file microorganisms-11-00971-s001.zip › Fig. S2.tif]
